# Maximal Standard Dose of Parenteral Iron for Hemodialysis Patients: An MRI-Based Decision Tree Learning Analysis

**DOI:** 10.1371/journal.pone.0115096

**Published:** 2014-12-15

**Authors:** Guy Rostoker, Mireille Griuncelli, Christelle Loridon, Théophile Magna, Philippe Janklewicz, Gilles Drahi, Hervé Dahan, Yves Cohen

**Affiliations:** 1 Division of Nephrology and Dialysis, Hôpital Privé Claude Galien, Générale de Santé, 20 route de Boussy, 91480 Quincy sous Sénart, France; 2 Division of Radiology, Hôpital Privé Claude Galien, Générale de Santé, 20 route de Boussy, 91480 Quincy sous Sénart, France; National Taiwan University Hospital, Taiwan

## Abstract

**Background and Objectives:**

Iron overload used to be considered rare among hemodialysis patients after the advent of erythropoesis-stimulating agents, but recent MRI studies have challenged this view. The aim of this study, based on decision-tree learning and on MRI determination of hepatic iron content, was to identify a noxious pattern of parenteral iron administration in hemodialysis patients.

**Design, Setting, Participants and Measurements:**

We performed a prospective cross-sectional study from 31 January 2005 to 31 August 2013 in the dialysis centre of a French community-based private hospital. A cohort of 199 fit hemodialysis patients free of overt inflammation and malnutrition were treated for anemia with parenteral iron-sucrose and an erythropoesis-stimulating agent (darbepoetin), in keeping with current clinical guidelines. Patients had blinded measurements of hepatic iron stores by means of T1 and T2* contrast MRI, without gadolinium, together with CHi-squared Automatic Interaction Detection (CHAID) analysis.

**Results:**

The CHAID algorithm first split the patients according to their monthly infused iron dose, with a single cutoff of 250 mg/month. In the node comprising the 88 hemodialysis patients who received more than 250 mg/month of IV iron, 78 patients had iron overload on MRI (88.6%, 95% CI: 80% to 93%). The odds ratio for hepatic iron overload on MRI was 3.9 (95% CI: 1.81 to 8.4) with >250 mg/month of IV iron as compared to <250 mg/month. Age, gender (female sex) and the hepcidin level also influenced liver iron content on MRI.

**Conclusions:**

The standard maximal amount of iron infused per month should be lowered to 250 mg in order to lessen the risk of dialysis iron overload and to allow safer use of parenteral iron products.

## Introduction

During the past two decades, routine use of erythropoeisis-stimulating agents (ESA) has enabled anemia to be corrected in most patients with end-stage renal disease, thereby improving their quality of life and reducing associated morbidity [1], [2]. Current clinical guidelines (KDOQI, KDIGO, and the European Best Practice Guideline) state that most dialysis patients receiving ESA should also receive parenteral iron supplementation to prevent iron deficiency [1]–[5]. Until recently, it was widely believed that iron overload among dialysis patients was more prevalent during the pre-ESA era, when blood transfusion was frequently used to treat anemia and when intravenous (IV) iron was given without concomitant ESA administration [1], [2], [5]. Iron overload was considered rare among dialysis patients in the ESA era [5].

We recently challenged this view, after studying liver iron stores by quantitative magnetic resonance imaging without gadolinium (MRI), the gold-standard method for estimating and monitoring iron stores [6], [7]. Indeed, we found that 84% of a cohort of 119 fit hemodialysis patients had hepatic iron overload on MRI (≥51 µmol/g dry weight), and that 30% of these 119 patients had severe iron overload (≥201 µmol/g dry weight) at levels usually seen in genetic hemochromatosis [6], [7]. Iatrogenic iron overload may therefore be prevalent among dialysis patients, favored in the USA and many other industrialized countries by recent implementation of reimbursement policies leading to an increase in the use of intravenous iron preparations aimed at reducing the cost of ESA, together with a possible excessive advocated dose of intravenous iron and, possibly, erroneous target values for biological markers of iron metabolism [6], [8], [9].

A strategy for safe use of intravenous iron products advocated by well-known physicians in the domain [10] was very recently endorsed by the KDIGO Controversies Conference which took place in San Francisco in late March 2014 [11]. The same KDIGO conference proposed a research agenda on dialysis iron overload [11].

We postulated that analysis of liver iron content (LIC) in dialysis patients by means of quantitative MRI, a new research tool that overcomes a major hypothetical limitation in hemodialysis patients, namely bone marrow iron depletion despite severe hepatosplenic siderosis, might, in combination with data-mining statistical methods, allow us to determine both a non toxic dose of infused iron and relevant target values for biological markers of iron metabolism, thereby improving the safety of parenteral iron products in dialysis patients [6], [7], [12].

The aim of this complementary study was to identify, by using decision-tree learning methodology based on the CHAID algorithm, a deleterious pattern of parenteral iron infusion. For this purpose we analysed the previous cohort of 119 fit hemodialysis patients studied by hepatic MRI [6], and 80 new patients studied in the same way.

## Materials and Methods

### Patients and dialysis

With the patients’ informed consent and ethical approval from the Drug, Devices and Clinical Trials Committee of our institution (COMEDIMS Claude Galien, December 2004), 213 fit patients with a dialysis vintage of at least 3 months, undergoing chronic intermittent bipuncture bicarbonate hemodialysis (with ultrapure dialysate and single-use biocompatible membranes) three times a week, were enrolled in this prospective cross-sectional study from 31 January 2005 to 31 August 2013. The exclusion criteria were as follows: refusal to participate in the study, poor compliance with the dialysis therapy schedule, age <18 years, severe cognitive impairment, claustrophobia, hepatic cirrhosis, overt inflammation (C-reactive protein >125 mg/L) or infectious disease, malnutrition (albuminemia <30 g/L), recent major bleeding (<3 months), major surgery (<3 months), transfusion dependency, recent transfusion (<3 months), intractable malignancy, cardiac pacemakers and metallic cardiac valves. The participants provided their written consent after having received verbal explanations by their nephrologist, together with a detailed information sheet. Signed informed consent forms were kept in a loose-leaf file. These procedures were approved by the Drug, Devices and Clinical Trials Committee of our institution. Of these 213 patients, 119 patients were the subject of a recent publication highlighting the risk of iron overload in this setting [6].

In keeping with the European Best Practice guideline, from the beginning of enrolments (31 January 2005) to 31 January 2010, anemia treatment in our hemodialysis centre comprised once-weekly intravenous administration of darbepoetin alpha and, if required, 100 mg of iron-sucrose (Venofer vial, 100 mg/5 ml Vifor International, Villars sur Glâne, Switzerland) starting twice to three times a week (induction phase), then once a week to once every four weeks (maintenance phase), with the following targets: hemoglobin 10–12 g/dL; transferrin saturation (TSAT): lower limit: 20%, target range 30%–50%; and serum ferritin: lower limit: 100 µg/L, target range: 200 to 500 µg/L [2]. Anemia treatment efficacy was estimated by hemoglobin assay and reticulocyte counts every 2 weeks, and by monthly measurements of biochemical markers of iron metabolism. The first 119 patients in the cohort were treated for anemia according to this schedule [6]. For the latest patients enrolled in the cohort, the results of our published study on the risk of iron overload and our call for a revision of guidelines in this area led us to anticipate the new European guideline which set upper targets for ferritin at 300 µg/L and TSAT at 30%, and a hemoglobin target of 10–12 g/dL [4], [6]. Moreover, for economic reasons, iron-sucrose Venofer was replaced in our hospital by a generic (iron-sucrose Actavis), as authorised by the European Medicines Agency (EMA).

15 patients screened for participation in the study were excluded, 12 for technical contra-indications to MRI (10 with a cardiac pacemaker; 1 for metallic debris in the eyes; 1 for claustrophobia), 1 for a severe inflammatory state related to chronic pelvic abscess, and 2 patients who repeatedly missed their MRI exam. Moreover, some relevant data on iron infusions were missing for 14 of the 94 patients included in the second cohort, who were thus excluded from the present analysis.

Medical records and dialysis charts, from dialysis initiation to the day of MRI, were carefully reviewed by a clinical research technician and checked by another clinical research technician, focusing on the volume of packed red blood cells (RBCs) transfused, iron infusions during dialysis sessions, and the dose of darbepoetin alpha. The iron doses received by patients are expressed in mg/month, which represents a median cumulative monthly dose. The methodology of this cross-sectional study, is described in depth in our recently published article [6].

### Quantitative magnetic resonance imaging (MRI) of hepatic iron stores

We used a signal-intensity-ratio method based on T1 and T2* contrast imaging without gadolinium, as established by Gandon and coworkers at Rennes University and validated in a cohort of 191 patients who underwent liver biopsy for biochemical iron assay [13]. Details of the MRI technique are described in our recently published article [6].

MRI measurements were made by four senior radiologists (PJ, YC, HD and GD) who were unaware of the patients’ medical history and biochemical results. Liver iron content was expressed in µmol/g of dry liver. As hepatic MRI can accurately detect liver iron overload exceeding 50 µmol/g, the upper limit of normal was set at 50 µmol/g for this study [6], [13]. Values between 51 and 100 µmol/g were considered to represent mild iron overload, values between 101 and 200 µmol/g moderate iron overload; and values ≥201 µmol/g severe iron overload [6], [13]. These LIC cutoffs are evidence-based and are based on previous and current data from liver biopsy and MRI; these gradual categories of iron overload reflect an increasing risk of complications in iron overload disorders such as genetic hemochromatosis and secondary hemosiderosis related to hematological disorders [7].

Patients on IV iron therapy received their last iron dose at least one week before MRI.

### Statistical Analyses

#### Descriptive statistics and comparison of the two patient cohorts

As values did not conform to a Gaussian distribution (Shapiro-Wilk normality test), all data are expressed as medians and ranges; percentages are given with their 95% confidence intervals calculated with the modified Wald method [14]. The two cohorts of patients were compared by using the non-parametric two-tailed Mann and Whitney test for continuous variables, and with the chi-square test for categorical variables [14]. To confirm a similar effect of Venofer and its generic Actavis on the risk of iron accumulation in the body, we sought correlations between MRI LIC values in these two cohorts of patients on the one hand, and, on the other hand, the cumulative iron dose infused in the year before MRI and the iron dose infused per month in the year before MRI, using Spearman’s rank-order correlation coefficient [14], [15]. Prism 6 software (Graphpad, San Diego, USA) was used for all tests, and p values<0.05 were considered to denote statistical significance [14].

#### Binary logistic regression

We applied standard binary logistic regression analysis (SPSS software from IBM, Bois-Colombes, Fance) to the whole cohort of 199 patients in order to determine the ability of several variables identified in our previous work, and other clinically relevant variables, to classify patients as having normal (≤50 µmol/g) or elevated (≥51 µmol/g) hepatic iron stores on MRI [6], [14]. This analysis included the three variables shown by binary regression logistic analysis to correlate with hepatic iron stores in the first 119 patients, namely the iron dose infused per month, and hepcidin and C-reactive protein levels [6]. We also included gender, as contingency tables in our previous work showed that the gender distribution differed strongly between patients with a normal liver iron content and those with iron overload [6]. The following seven clinically relevant variables were also included in the binary logistic regression analysis: ESA dose/month, red blood cells transfused per month, the dialysis vintage, Liu’s dialysis comorbidity index, age, Charlson’s index, and the audit score.

#### Decision-tree learning methodology

We used decision-tree learning based on CHAID (CHi-squared Automatic Interaction Detection) analysis with Bonferroni adjustment, implemented with SPSS software (IBM Bois-Colombes, France) [16]. We included in CHAID analysis the variables shown by the above binary logistic regression analysis to correlate with MRI hepatic iron stores [17]. The target variable was liver iron content, considered normal (≤50 µmol/g dry weight) or abnormal (≥51 µmol/g dry weight) by MRI [6], [13], [17]. The results of the CHAID algorithm were validated by cross-validation on the whole cohort of 199 patients [16].

## Results

### Characteristics of the study population

The patients’ demographic, clinical and biological characteristics are summarized in tables 1 and 2. The patients in the second cohort were older and more frequently diabetic, and had a lower dialysis vintage (Table 1). Interestingly, the lower ferritin and TSAT targets used in these patients had a substantial impact on their risk of iron overload: the overall percentage of patients with iron liver overload (LIC≥51 µmol/g) fell from 84% in the first cohort to 65% in the second cohort (p<0.005, Chi2 test; relative risk: 0.46, 95% CI: 0.27 to 0.76; odds ratio: 0.35, 95%CI: 0.18 to 0.69) (Table 1). Moreover, the risk of severe iron overload (LIC≥201 µmol/g) with potential clinical consequences also fell, from 30% to 11% (p<0.005, Chi2 test; relative risk: 0.37, 95% CI: 0.19 to 0.73; odds ratio: 0.29, 95% CI: 0.13 to 0. Both the cumulative dose of iron infused in the year before MRI (first cohort: rho = 0.31(0.07–0.52), p = 0.01; second cohort: rho = 0.37(0.03–0.63), p = 0.03; Spearman correlation test) and the iron dose infused per month in the year before MRI (first cohort: rho = 0.31(0.07–0.52), p = 0.01; second cohort: rho = 0.37(0.04–0.63), p = 0.03; Spearman correlation test) correlated closely with hepatic iron status on MRI, within the same range, strongly suggesting a similar risk of liver accumulation with the two iron-sucrose pharmaceuticals [15].

**Table 1:** Characteristics and findings in 199 hemodialysis patients studied by hepatic MRI to determine liver iron content (LIC).

Variables	Overall Cohort (n°1+n°2) n = 199	Cohort n°1 n = 119	Cohort n°2 n = 80	p value at Mann and Whitney test or Chi2test at comparison of cohort 1 and 2
Age (years)	64 [19–91]	60 [19–87]	70.50 [23–91]	p = 0.0005 at Mann and Whitney test
Female sex Percentage of patients (%)	38.69 [32.20–45.62]	38.66 [30.38–47.64]	38.75 [28.81–49.72]	p = 0.989 at X^2^ test
Dialysis vintage (months)	11 [2–95]	16 [2–95]	8.50 [2–66]	p = 0.0005 at Mann and Whitney
ESA Therapy Percentage of patients (%)	97.49 [94.09–99.09]	99.16 [94.93–99.99]	95 [87.45–98.42]	p = 0.169 at X^2^ test
Darbepoetin Dose (µg/month)	143 [0–775]	130 [0–566]	157.80 [0–775]	p = 0.0085 at Mann and Whitney test
Parenteral iron therapy, Percentage of patients (%)	90.95 [86.08–94.27]	94.96 [89.21–97.90]	85 [75.43–91.36]	p = 0.0316 at X^2^ test
Iron dose (mg/month)	225 [0–900]	169.20 [0–900]	303.20 [0–790]	p<0.0001 at Mann and Whitney test
Liu's dialysis Comorbidity index	3 [0–13]	3 [0–13]	3 [0–11]	p = 0.593 at Mann and Whitney test
Charlson's Comorbidity index	6 [2]–[16]	6 [2]–[16]	7 [2]–[16]	p = 0.0152 at Mann and Whitney test
Diabetes, Percentage of patients (%)	29.15 [23.26–35.82]	22.69 [16.04–31.05]	38.75 [28.81–49.72]	p = 0.0223 at X^2^ test
Audit Score (Alcohol Use Disorder Identification Test)	2 [0–40]	2 [0–40]	2 [1–40]	p = 0.379 at Mann and Whitney
Normal LIC at MRI, number of patients (%) (LIC≤50 µmol/g at MRI)	23.62 [18.23–30]	15.97 [10.38–23.68]	35 [25.43–45.94]	p = 0.0034 at X^2^ test
Mild hepatic iron overload at MRI, number of patients (%) (LIC: 51 to 100 µmol/g at MRI)	37.69 [31.24–44.60]	35.29 [27.28–44.23]	41.25 [31.10–52.20]	p = 0.483 at X^2^ test
Moderate hepatic iron overload at MRI, number of patients (%) (LIC: 101 to 200 µmol/g at MRI)	16.08 [11.59–21.86]	18.49 [12.47–26.48]	12.50 [6.74–21.69]	p = 0.352 at X^2^ test
Severe hepatic iron overload at MRI, number of patients (%) (LIC>200 µmol/g at MRI)	22.61 [17.33–28.93]	30.25 [22.70–39.04]	11.25 [5.82–20.23]	p = 0.0030 at X^2^ test

MRI: Magnetic Resonance Imaging LIC: Liver Iron Content.

Values are given as median and [range].

**Table 2:** Biochemical markers of iron metabolism in 199 hemodialysis patients studied by hepatic MRI to determine liver iron content (LIC).

Variables	Overall Cohort (n°1+n°2) n = 199	Cohort n°1 n = 119	Cohort n°2 n = 80	P Value at Mann and Whitney test or Chi2test at comparison of cohort 1 and 2
Hemoglobin (g/dL)	11.56 [8.38–15.12]	11.97 [8.43–15.12]	11.14 [8.38–14.68]	p = 0.0029 at Mann and Whitney test
C-reactive protein (mg/L)	4.15 [0.30–107.30]	4.30 [0.30–75.93]	3.93 [1–107.30]	p = 0.701 at Mann and Whitney test
Serum ferritin (µg/L)	205 [12–2229]	265.50 [15–1383]	145.30 [12–2229]	p = 0.0009 at Mann and Whitney test
Serum iron (µmol/l)	10.10 [3.59–26.27]	9.65 [3.59–26.27]	10.55 [4.18–26.27]	p = 0.280 at Mann and Whitney test
Serum transferrin (g/L)	1.80 [1.07–4.47]	1.69 [1.07–2.77]	1.95 [1.23–4.47]	p<0.0001 at Mann and Whitney test
Transferrin saturation (TSAT) (%)	22.60 [6.33–72.16]	23.07 [6.33–72.16]	21.63 [6.50–61.17]	p = 0.167 at Mann and Whitney test
Soluble transferrin receptor (sTfr) (mg/L)	4.89 [0.48–13.02]	4.27 [1.43–13.02]	5.44 [0.48–12.84]	p = 0.0038 at Mann and Whitney test
STfr/ferritin ratio	28.02 [0.22–1070]	21 [1.65–732.70]	34.04 [0.22–1070]	p = 0.0033 at Mann and Whitney test
Hepcidin (ng/ml)	51.87 [0.19–1036]	102.60 [0.76–1036]	29.86 [0.19–437.30]	p<0.0001 at Mann and Whitney test
Serum ferritin ≤100 µg/L (% patients)	26.63 [20.96–33.19]	21.01 [14.60–29.23]	35 [25.43–45.94]	p = 0.043 at X^2^ test
Serum ferritin ≥300 µg/L (% patients)	31.16 [25.12–37.91]	37.82 [29.60–46.79]	21.25 [13.62–31.52]	p = 0.020 at X^2^ test
Serum ferritin ≥500 µg/L (%patients)	15.58 [11.16–21.30]	18.49 [12.47–26.48]	11.25 [5.82–20.23]	p = 0.238 at X^2^ test
TSAT≥30% (%patients)	23.62 [18.23–30]	29.41 [21.95–38.16]	15 [8.64–24.57]	p = 0.030 at X^2^ test
TSAT≥50% (%patients)	4.52 [2.28–8.49]	3.36 [1.03–8.61]	6.25 [2.36–14.15]	p = 0.540 at X^2^ test

MRI: Magnetic Resonance Imaging.

Values are given as median and [range].

### Multiple logistic regression analysis

Binary logistic regression analysis showed that a combination of 4 variables correctly classified the hemodialysis patients as those with normal liver iron content and those with iron overload, namely the iron dose infused per month, age, gender, and the hepcidin level (Table 3).

**Table 3:** Correlations (binary logistic regression) between demographic and clinical continuous variables and biochemical markers and hepatic iron stores determined by MRI in 199 hemodialysis patients.

Parameter	Odds Ratio (and 95% CI)
Age (years)	OR = 0.948 (0.904–0.993); p = 0.025
Gender (Women/Men)	OR = 3.902 (1.378–11.052); p = 0.010
Dialysis vintage (months)	p = 0.089
Audit Score	p = 0.067
Liu's dialysis comorbidity Index	p = 0.295
Charlson's comorbidity Index	p = 0.227
Number RBC packstransfused×100/months of dialysis	p = 0.236
Darbopoetin dose per month	p = 0.418
Iron dose per month	OR = 1.007 (1.004–1.011); p = 0.000
C-reactive protein	p = 0.224
Hepcidin	OR = 1.008 (1.002–1.015); p = 0.007

CI: Confidence Interval; MRI: Magnetic Resonance Imaging; OR: Odds ratio; RBC: Red Blood Cell.

### Decision tree learning

**Figure 1:**
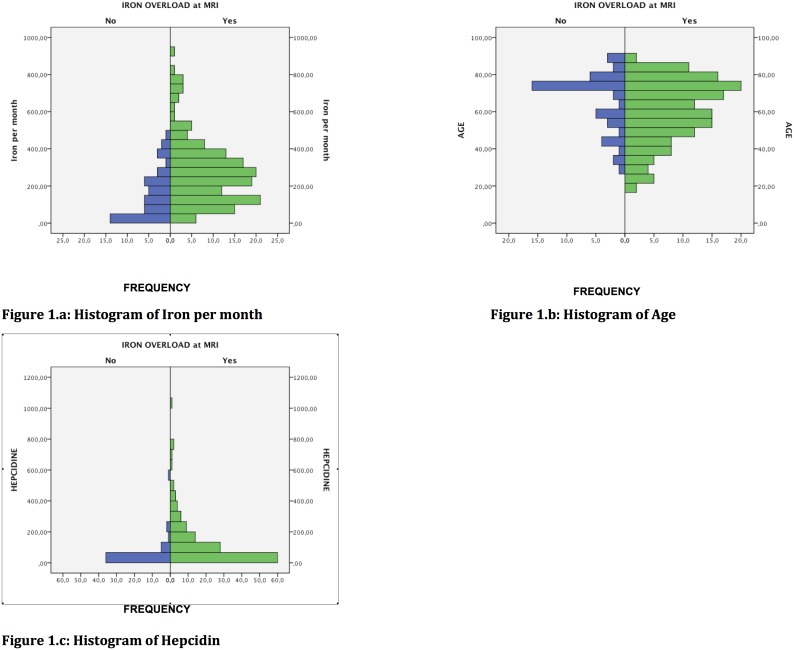
Histograms of monthly iron doses, age, and hepcidin levels in the overall cohort of 199 hemodialysis patients studied by MRI to determine hepatic iron content.

**Figure 2:**
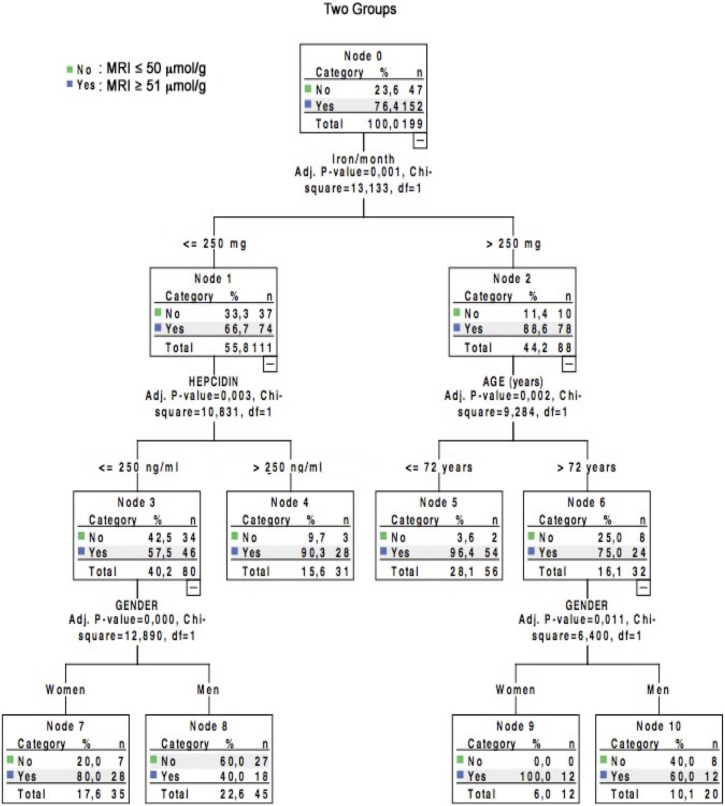
Tree diagram based on CHAID analysis in a cohort of 199 hemodialysis patients studied by MRI to determine liver iron content.

CHAID analysis with Bonferroni adjustment included the above 4 variables shown by binary logistic regression analysis to correlate with MRI hepatic iron stores (age, gender, iron dose per month and hepcidin). For the model, we chose an age cut-off of 72 years (2 categories) and a hepcidin cut-off of 250 ng/ml (2 categories), based on the histogram of the cohort values according to MRI hepatic iron status (Fig. 1). For the iron dose received per month, the algorithm comprised 3 categories (0 to 250 mg/month, between 250 mg/month and 500 mg/month, and more than 500 mg/month), based on epidemiological data and the histogram of the cohort values according to MRI hepatic iron status (Fig. 1) [9]. All 4 variables were considered relevant by the CHAID algorithm, and the classification tree comprised 10 nodes, 4 of which were terminal (Fig. 2, see also S1 Table). Very interestingly, the CHAID algorithm first split the patients exclusively according to their monthly infused iron dose, with a single cutoff of 250 mg/month (Fig. 2, see also S1 Table). The crude percentage of patients with iron overload on MRI (≥51 µmol/g dry weight) rose from 76.4% in the whole cohort to 88.6% in patients receiving more than 250 mg/month of IV iron (Fig. 2, see also S1 Table). In this node (n°2), which comprised 88 hemodialysis patients who received more than 250 mg/month of IV iron, 78 patients had iron overload on MRI (88%; 95% CI: 80% to 93%) (Fig. 2, see also S1 Table). The odds ratio for abnormal hepatic MRI in patients receiving more than 250 mg/month of IV iron as compared to those receiving less than 250 mg/month was 3.9 (95% CI: 1.81 to 8.4) (relative risk 2.93 (95% CI: 1.55 to 5.56)).

In hemodialysis patients who received less than 250 mg/month of IV iron, the hepcidin level had a powerful influence on hepatic iron stores, in keeping with its pathophysiological actions (Fig. 2, see also S1 Table). The CHAID algorithm was stable and reproducible and was cross-validated. It was also clinically relevant for patient classification according to liver iron status on MRI, correctly classifying 80.9% of the patients.

## Discussion

Our recently published cross-sectional study pointed to a role of the dose of infused iron in dialysis iron overload, a recently rediscovered and frequent clinical situation [6], [8], [18]–[20]. In addition, serial hepatic MRI examination of patients on parenteral iron therapy showed a clear relationship between the infused iron dose and liver iron content, while hepatic MRI monitoring after iron withdrawal further confirmed the deleterious role of parenteral iron therapy in this setting [6]. It should be noted that hepatic iron stores measured by MRI are a surrogate marker with yet unproven clinical relevance in dialysis patients, in terms of mortality and morbidity.

We therefore postulated that statistical methods used for data mining, coupled with LIC analysis by MRI, could help to determine both safe and deleterious IV iron doses for hemodialysis patients [6], [10], [11], [16]. Very interestingly, our present comparison of two cohorts of hemodialysis patients who differed only by their ferritin and TSAT target values showed a substantial impact of these values on the risk of iron overload. In the overall cohort, multivariable analysis confirmed the role of the monthly iron dose and the hepcidin level in the risk of dialysis iron overload, and extended our knowledge of the role of age and gender. In keeping with findings from Canavese's group and with evidence of a higher relative risk of iron overload in female patients based on contingency tables in our previous work, binary logistic regression in the current cohort clearly showed that iron homeostasis in hemodialysis patients is gender-related, with an odds ratio of 3.9 (95% CI: 1.38–11.05) in women versus men for abnormal liver iron content on MRI [6], [18].

The results of our CHAID analysis strongly suggest that an intravenous iron dose below 250 mg/month may reduce the risk of hepatic iron overload in hemodialysis patients and may contribute to safer use of parenteral iron.

This amount of IV iron of less than 250 mg/month is in keeping with data from Feldman and coworkers on the safety of a cumulative parenteral iron dose of 1000 mg over 6 months in hemodialysis patients, and with the recent finding that iron maintenance therapy at 200 mg/month is not associated with an increased short-term risk of infections, as encountered with bolus characterized by a monthly iron exposure of 700 mg [21]–[23]. Interestingly, the 250 mg/month cut-off dose of IV iron identified in the present study is lower than the two monthly doses of iron shown to be associated with higher death rates in hemodialysis patients: 300 mg in the recent Dialysis Outcomes and Practice Patterns Study and 400 mg in the Da Vita cohort study published eight years ago [24], [25].

This iron dose limit of 250 mg/month shown here to be safer with respect to the risk of iron overload is a median cumulative monthly dose which is close to the maintenance doses advocated by current guidelines, namely 88 to 260 mg/month in the American guideline (KDOQI 2006) [1], endorsed in 2009 by the European Renal Best Practice document [2], and 100 to 200 mg/month advocated by the British NICE guideline in 2006 [24]. However, it is noteworthy that these maintenance phases follow an induction phase comprising infusions of 1 g or 1.2 g of IV iron, possibly repeated once, as advocated by guidelines and labels [1]–[3], [5], [26]. It is also worthy of note that the recent KDIGO guideline did not advocate a particular maintenance IV iron dose but rather proposed TSAT (30%) and ferritin (500 µg/L) target values [3], and that these new target values for iron biomarkers were not endorsed by EDTA-ERA because of the potential risk of iron overload [4]. Likewise, the Japanese Society for Dialysis recently proposed that a minimal amount of IV iron (up to 650 mg in the induction phase) should be given to CKD patients only in case of true iron deficiency (ferritin <100 µg/L) and warned against maintenance intravenous iron therapy [27].

A recent epidemiological analysis of the management of anemia in hemodialysis patients in the USA, based on USRDS data, showed an increase in the use of IV iron, from 64% of patients in 2002 to 76% in 2008, together with an increase in the infused dose from 166 mg/month to 216 mg/month [28]. In addition, during the first year of hemodialysis, the usual monthly infused dose of iron was shown to be far higher, ranging from 270 mg to 305 mg [28]. A similar phenomenon was observed in our second cohort of patients who had a low dialysis vintage. Moreover, the change made to the ESA label by the Food and Drug Administration in June 2010 led to an increase in the percentage of US patients receiving IV iron from 57% (August 2010) to 71% (August 2011), together with a significant decline in the ESA dosage and an increase in the median ferritin level from 556 to 650 µg/L, with values exceeding 800 µg/L in 34% of patients [29]. While the median dose of IV iron remained largely stable at 190 mg/month, it is noteworthy that 18% of patients received more than 500 mg/month during this period [29]. Very similar trends in the use of IV iron were recently observed in other industrialised countries [9].

The main limitations of this study relate to its observational and cross-sectional design, and its execution in a single center in Europe. There is also a need for a lengthy, prospective, randomized trial comparing low, medium and high IV iron doses, with serial MRI (twice yearly) and an analysis of morbidity and mortality to endorse the findings reported here and in recent epidemiological studies.

## Conclusion

This study based on decision-tree learning with the CHAID algorithm in a cohort of 199 fit hemodialysis patients studied by MRI to determine hepatic iron stores and treated for anemia with ESA and IV iron strongly suggests that the standard maximal amount of iron infused per month should be 250 mg in order to reduce the risk of dialysis iron overload.

## Supporting Information

S1 Table **Individual data points on 199 hemodialysis patients studied by MRI, and CHAID decision-tree learning analysis of the maximal standard dose of parenteral iron.** French law defines these data as personal information in the possession of the patients' physicians; after extraction from the medical charts, they were rendered anonymous and an ID number was randomly attributed to each patient independently of age, gender, dialysis vintage and date of inclusion in the study.
(DOC)
