# Supplementary material for: Maximal Standard Dose of Parenteral Iron for Hemodialysis Patients: An MRI-Based Decision Tree Learning Analysis
Source: PLoS One. 2014 Dec 15;9(12):e115096. doi: 10.1371/journal.pone.0115096 (PMC4266677; doi:10.1371/journal.pone.0115096)
Supplement: S1 Table — Individual data points on 199 hemodialysis patients studied by MRI, and CHAID decision-tree learning analysis of the maximal standard dose of parenteral iron. French law defines these data as personal information in the possession of the patients' physicians; after extraction from the medical charts, they were rendered anonymous and an ID number was randomly attributed to each patient independently of age, gender, dialysis vintage and date of inclusion in the study. (DOC) [file pone.0115096.s001.doc]

**Supporting Information**

**Table S1: Individual data points on 199 hemodialysis patients studied by MRI, and CHAID decision-tree learning analysis of the maximal standard dose of parenteral iron.**

French law defines these data as personal information in the possession of the patients' physicians; after extraction from the medical charts, they were rendered anonymous and an ID number was randomly attributed to each patient independently of age, gender, dialysis vintage and date of inclusion in the study.

| **LIC at MRI micromol/g** | **Age (Years)** | **Gender (Male= 0; female=1)** | **Iron dose (mg/month)** | **Hepcidine (ng/ml)** |
| --- | --- | --- | --- | --- |
| 65 | 52 | 1 | 224,07 | 105,72 |
| 340 | 74 | 1 | 169,18 | 51,87 |
| 60 | 59 | 0 | 354,5 | 4,53 |
| 20 | 59 | 0 | 200 | 3,04 |
| 90 | 47 | 1 | 138,82 | 144,93 |
| 230 | 69 | 0 | 217,09 | 770,12 |
| 150 | 84 | 1 | 272,7 | 51,38 |
| 90 | 60 | 0 | 114,29 | 51,87 |
| 65 | 69 | 0 | 271,43 | 7,7 |
| 30 | 32 | 0 | 0 | 29,86 |
| 95 | 51 | 0 | 300 | 30,29 |
| 100 | 43 | 0 | 416,7 | 75,77 |
| 40 | 50 | 1 | 135 | 554,78 |
| 5 | 41 | 0 | 0 | 10,74 |
| 100 | 42 | 1 | 225 | 51,87 |
| 270 | 75 | 0 | 50 | 86,35 |
| 70 | 76 | 0 | 97,44 | 51,87 |
| 270 | 61 | 0 | 84,21 | 408,81 |
| 30 | 36 | 0 | 126,19 | 44,02 |
| 5 | 42 | 0 | 150 | 3,68 |
| 160 | 33 | 0 | 187,5 | 250,21 |
| 65 | 49 | 0 | 220 | 47,25 |
| 5 | 74 | 0 | 77,8 | 5,77 |
| 210 | 67 | 1 | 129,79 | 473,33 |
| 45 | 78 | 0 | 165,31 | 51,87 |
| 300 | 71 | 0 | 221,43 | 779,85 |
| 60 | 35 | 0 | 0 | 15,25 |
| 120 | 44 | 1 | 330 | 144,57 |
| 50 | 75 | 0 | 57,14 | 174,84 |
| 70 | 46 | 1 | 333,33 | 20,66 |
| 200 | 37 | 0 | 290 | 36,36 |
| 160 | 77 | 0 | 380 | 10,84 |
| 15 | 76 | 0 | 267,9 | 110,61 |
| 40 | 89 | 0 | 262,5 | 13,03 |
| 270 | 58 | 0 | 72,73 | 141,61 |
| 290 | 59 | 1 | 152,5 | 237,63 |
| 310 | 52 | 1 | 343,16 | 1035,5 |
| 60 | 42 | 0 | 150,58 | 6,98 |
| 200 | 43 | 1 | 265,28 | 1,23 |
| 40 | 65 | 0 | 361,5 | 56,83 |
| 220 | 72 | 0 | 283,33 | 51,87 |
| 250 | 47 | 1 | 108,9 | 274,64 |
| 250 | 55 | 1 | 82,43 | 206,52 |
| 170 | 69 | 0 | 650 | 5,04 |
| 220 | 82 | 0 | 900 | 59 |
| 45 | 78 | 0 | 174,29 | 52,33 |
| 250 | 76 | 0 | 102,75 | 516,83 |
| 77 | 44 | 1 | 129,17 | 234 |
| 120 | 78 | 0 | 466,67 | 211,89 |
| 240 | 84 | 0 | 58,97 | 51,87 |
| 5 | 72 | 0 | 100 | 16,18 |
| 300 | 73 | 1 | 0 | 337,84 |
| 65 | 74 | 1 | 110,29 | 11,11 |
| 50 | 75 | 0 | 123,53 | 218,95 |
| 100 | 23 | 0 | 523,1 | 3,65 |
| 200 | 35 | 0 | 347,1 | 2,85 |
| 300 | 84 | 0 | 223,08 | 51,87 |
| 330 | 65 | 0 | 816,67 | 51,87 |
| 85 | 70 | 0 | 383,3 | 85,96 |
| 60 | 81 | 0 | 440 | 22,33 |
| 220 | 79 | 0 | 39,47 | 141,3 |
| 310 | 67 | 0 | 175 | 113,7 |
| 90 | 63 | 0 | 165,38 | 51,87 |
| 90 | 79 | 1 | 200 | 6,53 |
| 30 | 52 | 0 | 0 | 44,35 |
| 320 | 73 | 1 | 564,7 | 437,26 |
| 65 | 31 | 1 | 300 | 42,6 |
| 40 | 29 | 0 | 133,33 | 40,71 |
| 65 | 85 | 1 | 342,9 | 4,84 |
| 230 | 58 | 0 | 278,79 | 6,94 |
| 5 | 44 | 1 | 0 | 4,29 |
| 60 | 87 | 0 | 400 | 87,88 |
| 100 | 71 | 0 | 450 | 14,11 |
| 240 | 68 | 0 | 306,3 | 35,74 |
| 70 | 52 | 0 | 400 | 2,32 |
| 60 | 52 | 1 | 0 | 1,01 |
| 40 | 77 | 1 | 225 | 5,44 |
| 25 | 81 | 0 | 198,15 | 62,94 |
| 240 | 52 | 0 | 130,52 | 311,22 |
| 210 | 25 | 0 | 370 | 51,41 |
| 70 | 64 | 1 | 166,7 | 14,32 |
| 160 | 53 | 1 | 233,33 | 1,31 |
| 40 | 78 | 0 | 300 | 31,96 |
| 230 | 52 | 1 | 355,9 | 44,43 |
| 170 | 29 | 0 | 242,86 | 145,28 |
| 90 | 29 | 1 | 50 | 22,83 |
| 290 | 39 | 0 | 335,29 | 681,63 |
| 55 | 75 | 1 | 381,8 | 0,87 |
| 5 | 44 | 0 | 0 | 39 |
| 65 | 58 | 0 | 363,5 | 51,87 |
| 25 | 85 | 0 | 450 | 0,96 |
| 85 | 52 | 1 | 255,8 | 118,04 |
| 230 | 59 | 1 | 371,43 | 47,34 |
| 80 | 85 | 0 | 171,4 | 364,54 |
| 55 | 64 | 0 | 340 | 13,08 |
| 70 | 60 | 0 | 136,18 | 421,41 |
| 250 | 76 | 0 | 115,63 | 325,98 |
| 85 | 70 | 0 | 122,22 | 103,02 |
| 260 | 52 | 0 | 80,32 | 162,74 |
| 5 | 43 | 0 | 0 | 15,57 |
| 100 | 62 | 0 | 350 | 51,87 |
| 70 | 66 | 1 | 235,7 | 74,02 |
| 55 | 53 | 0 | 400 | 33,89 |
| 60 | 69 | 1 | 84,78 | 103,99 |
| 70 | 26 | 0 | 361,5 | 32,53 |
| 40 | 68 | 0 | 0 | 43,84 |
| 70 | 71 | 1 | 140 | 212,4 |
| 150 | 55 | 1 | 128,57 | 105,85 |
| 70 | 21 | 0 | 300 | 51,87 |
| 90 | 81 | 1 | 150 | 14,2 |
| 5 | 55 | 0 | 0 | 1,56 |
| 230 | 51 | 1 | 666,7 | 14,86 |
| 70 | 80 | 1 | 0 | 124,38 |
| 5 | 82 | 0 | 0 | 40,97 |
| 190 | 76 | 0 | 291,18 | 51,87 |
| 200 | 56 | 0 | 366,67 | 54,37 |
| 220 | 81 | 0 | 100 | 5,29 |
| 5 | 74 | 0 | 366,7 | 2,44 |
| 150 | 84 | 1 | 300 | 222,77 |
| 270 | 66 | 0 | 253,19 | 87,29 |
| 95 | 55 | 0 | 250 | 53,7 |
| 65 | 71 | 1 | 134,62 | 51,87 |
| 200 | 25 | 1 | 200 | 119,65 |
| 70 | 81 | 0 | 700 | 278,17 |
| 5 | 61 | 1 | 0 | 51,87 |
| 10 | 52 | 1 | 233,3 | 11,45 |
| 95 | 68 | 0 | 482,9 | 244,9 |
| 25 | 60 | 0 | 33,33 | 216,21 |
| 210 | 44 | 1 | 427,27 | 51,87 |
| 5 | 74 | 0 | 94,55 | 82,06 |
| 5 | 88 | 1 | 0 | 4,55 |
| 70 | 37 | 0 | 325 | 9,06 |
| 90 | 72 | 0 | 206,25 | 323,42 |
| 65 | 77 | 1 | 700 | 169,98 |
| 60 | 84 | 1 | 260 | 7,79 |
| 5 | 75 | 1 | 98,91 | 100 |
| 55 | 75 | 0 | 0 | 51,87 |
| 220 | 47 | 0 | 262,5 | 51,87 |
| 290 | 78 | 0 | 218,2 | 107,94 |
| 95 | 75 | 1 | 98,08 | 198,86 |
| 230 | 63 | 1 | 162,5 | 149,64 |
| 200 | 27 | 1 | 293,94 | 132,05 |
| 180 | 74 | 0 | 385,7 | 30,6 |
| 20 | 87 | 1 | 222,6 | 20,72 |
| 300 | 82 | 0 | 338,1 | 12,89 |
| 55 | 59 | 0 | 57,69 | 136,36 |
| 100 | 80 | 0 | 288,9 | 151,54 |
| 45 | 74 | 0 | 50 | 9,53 |
| 270 | 63 | 0 | 144,44 | 51,87 |
| 35 | 61 | 0 | 0 | 66,81 |
| 170 | 51 | 1 | 228,57 | 24,25 |
| 5 | 75 | 0 | 150 | 5,67 |
| 200 | 73 | 1 | 287,65 | 153,71 |
| 150 | 61 | 0 | 757,1 | 126,53 |
| 190 | 50 | 0 | 285,71 | 1,1 |
| 80 | 86 | 1 | 344,4 | 42,28 |
| 190 | 49 | 1 | 766,7 | 9,98 |
| 100 | 91 | 1 | 290 | 35,84 |
| 10 | 73 | 0 | 400 | 3,61 |
| 60 | 76 | 1 | 464,7 | 4,41 |
| 180 | 68 | 1 | 137,21 | 11,64 |
| 170 | 59 | 0 | 129,41 | 87,9 |
| 260 | 54 | 1 | 790 | 27,15 |
| 40 | 67 | 0 | 234,8 | 0,19 |
| 40 | 79 | 0 | 400 | 3,78 |
| 60 | 76 | 0 | 173,91 | 111,7 |
| 210 | 37 | 1 | 136,05 | 115,28 |
| 45 | 76 | 0 | 366,7 | 18,92 |
| 100 | 51 | 1 | 240 | 82,18 |
| 160 | 72 | 1 | 242 | 67,64 |
| 70 | 69 | 0 | 200 | 123,6 |
| 95 | 78 | 0 | 275 | 235,34 |
| 250 | 41 | 0 | 231,25 | 634,42 |
| 210 | 34 | 1 | 440 | 30,06 |
| 170 | 19 | 0 | 366,67 | 13,66 |
| 50 | 72 | 0 | 50 | 0,76 |
| 65 | 47 | 1 | 216,67 | 285,89 |
| 190 | 40 | 1 | 509,1 | 143,59 |
| 160 | 63 | 1 | 706,3 | 41,2 |
| 15 | 74 | 0 | 120,59 | 3,7 |
| 65 | 69 | 1 | 258 | 28,35 |
| 55 | 73 | 1 | 96,67 | 176,47 |
| 45 | 59 | 0 | 261,9 | 52,32 |
| 95 | 40 | 1 | 99,46 | 51,87 |
| 270 | 33 | 0 | 516,67 | 51,87 |
| 180 | 24 | 1 | 500 | 4,53 |
| 220 | 86 | 1 | 620 | 385,13 |
| 85 | 38 | 1 | 55,68 | 105,26 |
| 5 | 72 | 0 | 0 | 103,15 |
| 95 | 57 | 1 | 144,44 | 102,55 |
| 85 | 78 | 0 | 140,7 | 354,63 |
| 55 | 77 | 0 | 182,22 | 58,33 |
| 40 | 74 | 1 | 217,2 | 7,64 |
| 200 | 79 | 0 | 280 | 101,1 |
| 70 | 58 | 0 | 310,53 | 50,9 |
| 200 | 47 | 1 | 418,18 | 123,6 |
| 90 | 63 | 1 | 511,8 | 114 |
| 60 | 63 | 0 | 340 | 29,08 |
| 70 | 57 | 0 | 50 | 51,87 |
